# Circulating biomarkers and outcomes from a randomised phase 2 trial of gemcitabine versus capecitabine-based chemoradiotherapy for pancreatic cancer

**DOI:** 10.1038/s41416-020-01120-z

**Published:** 2020-10-26

**Authors:** Frances Willenbrock, Catrin M. Cox, Eileen E. Parkes, Charlotte S. Wilhelm-Benartzi, Aswin G. Abraham, Robert Owens, Ahmad Sabbagh, Christopher M. Jones, Daniel L. I. Hughes, Tim Maughan, Christopher N. Hurt, Eric E. O’Neill, Somnath Mukherjee

**Affiliations:** 1 Department of Oncology, University of Oxford, Oxford, UK; 2 Centre for Trials Research, Cardiff, UK; 3 Cross Cancer Institute, Edmonton, AB Canada; 4 Oxford University Hospital NHS Trust, Oxford, UK; 5 Weston Park Cancer Centre, Sheffield Teaching Hospitals NHS Foundation Trust, Sheffield, UK; 6 University of Leeds & Leeds Cancer Centre, Leeds, UK

**Keywords:** Pancreatic cancer, Tumour biomarkers

## Abstract

**Background:**

The Phase 2 SCALOP trial compared gemcitabine with capecitabine-based consolidation chemoradiotherapy (CRT) in locally advanced pancreatic cancer (LAPC).

**Methods:**

Thirty-five systematically identified circulating biomarkers were analysed in plasma samples from 60 patients enroled in SCALOP. Each was measured in triplicate at baseline (prior to three cycles of gemcitabine-capecitabine induction chemotherapy) and, for a subset, prior to CRT. Association with overall survival (OS) was determined using univariable Cox regression and optimal thresholds delineating low to high values identified using time-dependent ROC curves. Independence from known prognostic factors was assessed using Spearman correlation and the Wilcoxon rank sum test prior to multivariable Cox regression modelling including independent biomarkers and known prognostic factors.

**Results:**

Baseline circulating levels of C-C motif chemokine ligand 5 (CCL5) were significantly associated with OS, independent of other clinicopathological characteristics. Patients with low circulating CCL5 (CCL5^low^) had a median OS of 18.5 (95% CI 11.76–21.32) months compared to 11.3 (95% CI 9.86–15.51) months in CCL5^high^; hazard ratio 1.95 (95% CI 1.04–8.65; *p* = 0.037).

**Conclusions:**

CCL5 is an independent prognostic biomarker in LAPC. Given the known role of CCL5 in tumour invasion, metastasis and the induction of an immunosuppressive micro-environment, targeting of CCL5-mediated pathways may offer therapeutic potential in pancreatic cancer.

**Clinical trial registration:**

The SCALOP trial was registered with ISRCTN, number 96169987 (registered 29 May 2008).

## Background

Pancreatic cancer is a leading cause of cancer-related death and is predicted to be the second largest cause of death from cancer worldwide by the end of the current decade.^1^ Inoperable, non-metastatic, locally advanced pancreatic cancer (LAPC) has an intermediate prognosis (median overall survival (OS) 12–18 months) between metastatic and operable disease, and is treated with combination chemotherapy or chemoradiotherapy (CRT). The use of potent combination chemotherapy has delivered modest improvements in clinical outcomes, though its application in clinical practice is limited by toxicity.^2,3^ Recently, a greater understanding of tumour biology has driven the development of novel therapies.^4–6^ One example is the recently reported POLO trial of maintenance olaparib in patients with germline BRCA mutations, which is the first successful trial of biomarker-guided therapy in pancreatic cancer.^7^

Tissue and blood samples collected during clinical trials provide an unparalleled resource from which tumour biology can be further interrogated and correlated with clinical outcomes, thereby facilitating the generation of evidence-based hypotheses for biomarker-driven treatments. SCALOP was a multi-centre randomised Phase 2 trial (*n* = 114) which compared gemcitabine versus capecitabine as the radiosensitiser of choice for LAPC.^8^ Patients received three cycles of gemcitabine and capecitabine (GEMCAP) induction chemotherapy and eligible patients with responding or stable disease (*n* = 74) were randomised to a further cycle of GEMCAP followed by gemcitabine or capecitabine-based CRT to a total radiation dose of 50.4 Gy in 28 fractions. Survival analyses have previously been reported and demonstrate that capecitabine-based CRT was superior in terms of survival and toxicity profile.^8,9^ To facilitate later biomarker analysis, blood samples were collected from individual patients at baseline (prior to GEMCAP) and, for a subset, at week 17 (prior to consolidation CRT), as summarised in Supplementary Fig. 1.

In a prior systematic review, we identified a number of cytokines reported to be of diagnostic, prognostic or predictive significance in PDAC.^10^ Here, we report the results of a detailed prognostic biomarker analysis, including correlation with clinical outcomes, assessing a panel of 35 of these systematically identified cytokines in samples from the SCALOP trial cohort.

## Methods

This study report aligns with the Reporting Recommendations for Tumour Marker prognostic studies (REMARK) criteria.^11^

### Patients

The design and results of the SCALOP trial have been reported elsewhere.^8,9^ Briefly, patients with histologically/cytologically confirmed inoperable locally advanced pancreatic cancer with maximum diameter 7 cm or less, performance status (PS) 0–2, were eligible. Response was assessed following three cycles of GEMCAP chemotherapy, and those with responding or stable disease (according to RECIST criteria), PS 0–1 and tumour diameter 6 cm or less were eligible for randomisation to a further cycle of GEMCAP followed by gemcitabine (300 mg/m^2^ weekly) or capecitabine (830 mg/m^2^ twice daily on days of radiotherapy) concurrent with radiotherapy (50.4 Gy in 28 fractions), as summarised in Supplementary Fig. 1.

### Sample collection and analysis

Peripheral venous blood was collected in ethylenediaminetetraacetic acid (EDTA) anti-coagulant vacutainers. Each was centrifuged at 3000×*g* for 10 minutes and plasma aliquoted into cryovials then snap frozen. Samples were stored at −80 °C at investigating centres until the end of the trial and subsequently shipped on dry ice to Wales Cancer Bank (WCB) for centralised storage. Centres that were unable to store frozen blood samples or which did not have a Human Tissue Authority (HTA) license were required to send samples to WCB for processing using pre-paid safe boxes. All samples were processed on the same day of collection and, where possible, within an hour of collection.

Biomarker analysis was undertaken at the molecular laboratory at Oxford Institute for Radiation Oncology, University of Oxford. Assays were performed while blinded to clinical outcome. Assayed cytokines are listed in Table 1. Insulin-like growth factor 1 (IGF-1), transforming growth factor-β (TGF-β) and β-nerve growth factor precursor (β-NGF) were quantified using DuoSet® enzyme-linked immunosorbent assay (ELISA) (R&D Systems, USA) and the signal detected using a POLARstar® Omega microplate reader. The remaining 32 cytokines were assessed by Human Multiplexed Magnetic Luminex assay (R&D Systems, USA) and measured using the Luminex MAGPIX® fluorescent detection system.

**Table 1:** Association of each biomarker with overall survival.

Biomarker	*n*	Value (pg/ml)					*p*	FDR
		Mean	SD	95% CI	Median	IQR		
Beta-nerve growth factor (bNGF)	29	18.6	33.8	−47.6, 84.7	6.0	3.15, 14.0	0.192	0.8295
C-C motif chemokine ligand 11 (CCL11)	60	217.4	178.7	−132.8, 567.5	159.2	98.2, 257.2	0.5539	0.8858
C-C motif chemokine ligand 2 (CCL2)	60	240.8	135.4	−24.6, 506.0	209.0	154.9, 310.9	0.3209	0.8295
C-C motif chemokine ligand 27 (CCL27)	60	629.4	212.6	212.7, 1046.0	611.8	517.3, 746.7	0.8368	0.9414
C-C motif chemokine ligand 4 (CCL4)	60	596.5	121.3	358.7, 834.0	599.8	532.5, 746.7	0.3164	0.8295
C-C motif chem\okine ligand 5 (CCL5)	60	37205.9	24871.1	−11541.3, 85953.2	30108.5	20941.1, 48637.9	0.0133	0.1772*
C-C motif chemokine ligand 7 (CCL7)	60	117.8	39.9	49.7, 195.9	114.3	86.9, 141.7	0.7038	0.8858
Interleukin-2 receptor alpha chain (CD25/IL2RA)	60	1156.2	416.6	339.8, 1972.6	1093.6	843.6, 1373.2	0.2481	0.8295
C-X-C motif chemokine ligand 1 (CXCL1)	44	177.4	90.3	0.4, 354.4	167.1	127.3, 213.7	0.8965	0.9449
C-X-C motif chemokine ligand 10 (CXCL10)	60	63.4	50.5	−35.5, 162.3	50.8	5.8, 74.1	0.4322	0.8295
C-X-C motif chemokine ligand 8 (CXCL8)	60	152.6	403.2	−637.5, 942.8	17.8	9.7, 72.5	0.2613	0.8295
C-X-C motif chemokine ligand 9 (CXCL9)	60	712.5	174.6	370.4, 1054.5	670.9	616.7, 773.5	0.6452	0.8858
Granulocyte colony stimulating factor (G-CSF)	60	17.8	11.7	−5.2, 40.7	16.2	12.6, 18.9	0.8654	0.9414
Hepatocyte growth factor (HGF)	60	442.8	364.7	−271.9, 1157.5	381.4	60.1, 548.0	0.4276	0.8295
Intracellular adhesion molecule 1 (ICAM1)	60	998988.5	532408.5	−44532.5, 2042510	879960.3	704481.1, 1065006.5	0.9861	0.9882
Interferon γ (IFNγ)	60	87.6	124.3	−156.0, 331.1	69.2	55.4, 84.9	0.0136	0.1772*
Insulin-like growth factor-1 (IGF-1)	46	398.4	984.7	−1631.6, 2328.4	156.4	84.0, 279.6	0.7268	0.8858
Interleukin-10 (IL-10)	60	4.6	3.2	−1.6, 10.7	4.1	2.9, 4.6	0.4466	0.8295
Interleukin-13 (IL-13)	60	782.2	172.0	445.2, 1119.2	766.4	624.6, 940.6	0.7106	0.8858
Interleukin-16 (IL-16)	60	1905.9	2163.1	’−2333.7, 6145.4	1149.3	139.5, 3354.6	0.2311	0.8295
Interleukin-18 (IL-18)	60	2405.4	3401.7	−4261.8, 9072.6	1133.9	348.1, 3253.5	0.5726	0.8858
Interleukin-1β (IL-1β)	60	45.3	44.7	−42.2, 132.8	30.4	18.5, 55.5	0.4451	0.8295
Interleukin 1 receptor antagonist (IL-1RA)	60	6762.5	7899.3	−8720.0, 22245.0	4510.9	894.4, 11339.7	0.4949	0.8774
Interleukin-3 (IL-3)	60	57.4	42.9	−188.0, 335.5	50.7	32.8, 70.9	0.0616	0.8858
Interleukin-4 (IL-4)	60	74.8	20.9	33.8, 115.6	75.2	59.2, 90.4	0.442	0.8295
Interleukin-6 (IL-6)	60	7.2	5.1	−2.7, 17.0	5.7	4.9, 8.85	0.7172	0.8858
Interleukin-7 (IL-7)	60	8.2	3.5	1.3, 15.0	7.7	6.0, 9.3	0.3565	0.8295
Macrophage colony stimulating factor (M-CSF)	60	952.2	1065.2	1135.6, 3039.9	553.0	416.1, 1069.8	0.2069	0.8295
Macrophage inhibitory factor (MIF)	60	44434.9	69633.7	−92047.2, 180926.9	25334.0	7080.3, 50172.5	0.2781	0.8295
Osteopontin (OPN)	60	59412.9	20243.5	19735.7, 99090.0	59664.0	46423.2, 72998.8	0.869	0.9414
Platelet-derived growth factor (PDGF)	60	3790.5	2508.2	−1125.5, 8706.6	3535.8	1986.7, 5284.2	0.3548	0.8295
Stem cell factor (SCF)	60	92.6	31.2	31.4, 153.7	90.7	74.2, 106.8	0.1321	0.8295
Transforming growth factor-β (TGF-β)	46	32.4	16.5	−0.0, 64.7	31.5	21.7, 41.8	0.2677	0.8295
TNF-related apoptosis-inducing ligand (TRAIL)	60	65.9	47.4	−27.0, 158.7	54.3	38.3, 85.1	0.6925	0.8858
Vascular cell adhesion protein 1 (VCAM-1)	60	1381170.6	598701.2	207716.2, 2554625.0	1289218.1	1022870.6, 1562745.6	0.5633	0.8858
Vascular endothelial growth factor-A (VEGF-A)	48	92.2	77.0	−58.6, 243.0	68.9	0, 119.7	0.6493	0.8858
Week 17								
Beta-nerve growth factor (bNGF)	50	11.8	5.2	1.65, 22.0	10.8	7.7, 14.3	0.9882	0.9882
C-C motif chemokine ligand 5 (CCL5)	47	80240.0	86870.0	−90030, 250510	50620.0	33360, 92560	0.847	
Insulin-like growth factor-1 (IGF-1)	49	1066.3	2390.4	−3618.8, 5751.4	337.5	131.0, 616.7	0.4341	0.8295
Transforming growth factor-β (TGF-β)	50	12.9	9.1		9.9		0.8377	0.9414

Results are shown for baseline for all biomarkers and additionally for week 17 for an additional subset. Association with overall survival was determined using univariable Cox regression. Biomarkers significantly associated with survival at the *q* < 0.5 level are highlighted by “*”.

*FDR* false discovery rate, *n* number.

Prior to assay, all plasma samples were thawed on ice and centrifuged at 10,000×*g* for 5 min at 4 °C to remove precipitate. Samples were limited to two freeze-thaw cycles. Supernatants were loaded onto ELISA or Luminex plates at the recommended dilutions (i.e. 50-fold for CCL-5 and PDGF, each at 1 µl per well, and two-fold for all other cytokines, which were loaded at 25 µl per well) with standard protein controls. The manufacturer’s protocol was followed in each case with no modifications. For ELISA assays, standard curves were constructed and used to quantify each marker. For Luminex assays, biomarker quantification was derived from analysis of raw data using xPONENT 4.2® software.

All biomarkers were measured in triplicate across all patients at each timepoint. If a run contained ≥50% out of range values for a given biomarker across all patients (>1 SD from the mean) then the entire run was removed for that biomarker. Any remaining values above the upper limit of detection or below the lower limit of detection were substituted with the highest or half the lowest value of that biomarker respectively for a given run. The mean value across the remaining runs was then used for analysis. This method for handling measurement errors has been used elsewhere.^12^

### Statistical analysis

Statistical analyses were conducted using R v3.5.2 according to a pre-specified analysis plan. Univariate Cox proportional hazard models were used for each of the 35 biomarkers as continuous variables to determine the association with OS. OS was measured from randomisation until death from any cause. Multiple comparisons were accounted for by using the False Discovery Rate (FDR). Those found to be significant at the q value <0.2 were then further investigated for independence from existing prognostic clinical characteristics (i.e. cancer antigen 19–9 (CA19–9), treatment (capecitabine CRT vs gemcitabine CRT), PS (0 vs 1) and age (<65 vs ≥65 years)) using Spearman correlations (*r* ≤ 0.7 shows independence) and Wilcoxon rank sum tests (*p* ≥ 0.05 shows independence). PET-CT data were unfortunately unavailable for a majority of patients and could not therefore be included in these analyses. Those clinical characteristics found to be independent were then split into tertiles and associated with OS using univariate Cox regression to ensure that any associations with the biomarker as a continuous variable were linear. Optimal thresholds delineating low to high values were identified using the R “survivalROC” package based on time-dependent receiver operating characteristic (ROC) curves from censored survival data and their corresponding area under the curve (AUC). Both continuous and dichotomised biomarkers were then associated with OS using multivariable Cox proportional hazard models, along with existing prognostic clinical characteristics, to determine whether or not novel biomarkers maintained prognostic value. The proportional hazards assumption was tested by calculating Schoenfeld residuals, and the linearity assumption was assessed by plotting deviance residuals.

## Results

### Patient characteristics

Cytokine data were available from 63 patients in total. No significant differences in clinicopathological characteristics were identified comparing patients who did and did not have cytokine data available (Supplementary Table 1). Measurements of the full panel of cytokines and corresponding clinical outcome information were available for 60 patients. These data were assessed further in order to identify correlations between circulating cytokines and clinical outcomes.

### Prognostic factor identification

Raw biomarker levels at both baseline and, where measured, at week 17 are shown in Table 1, as is an analysis of the association with OS of each of the 35 assayed cytokines. Of the biomarkers tested, two (C-C Motif Chemokine Ligand 5, CCL5 and interferon-ƴ, IFNy) measured at baseline (prior to commencement of GEMCAP chemotherapy) had significant associations with OS at the *q* < 0.2 FDR level. IFNy levels significantly correlated with age (*p* = 0.019). However, CCL5 was independent of existing clinical characteristics, including age (*p* = 0.859), PS (*p* = 0.660) and CA19–9 (*r* = 0.339) (Table 2). No biomarkers were associated with progression at the univariate level at the *q* < 0.2 FDR level, as illustrated in Supplementary Table 2. Consequently, no further analyses on progression were undertaken.

**Table 2:** Correlation between biomarkers significantly associated with overall survival by Cox univariable regression and clinical characteristics known to be associated with overall survival in pancreatic cancer.

	CCL5		IFNy	
	Median (IQR) μg/ml	*p*-value	Median (IQR) pg/ml	*p*-value
Age (years)				
<65 (*n* = 33)	34.0 (19.2, 49.8)	0.859	59 (51.5, 79)	0.019
≥65 (*n* = 27)	28.2 (21.4, 45.8)		79 (62, 92.5)	
WHO PS				
0 (*n* = 34)	29 (21, 45.6)	0.660	151 (109, 168)	0.929
1 (*n* = 26)	32.4 (21, 51)		127 (113, 172)	
Treatment				
Capecitabine (*n* = 33)	33.6 (22.8, 49)	0.438	54 (31.4, 76)	0.679
Gemcitabine (*n* = 27)	28.2 (18, 47)		50.5 (39.5, 67.5)	
CA19–9 (*n* = 54)	*r* = 0.339	0.012	*r* = 0.012	0.9337
Longest disease diameter (*n* = 58)	*r* = 0.091	0.498	*r* = −0.087	0.5160

*CA19–9* cancer antigen 19–9, *CCL5* C-C chemokine ligand 5, *IFNƴ* interferon-ƴ, *IQR* interquartile range, *WHO PS* World Health Organisation Performance Status.

### CCL5 is an independent prognostic biomarker in LAPC

CCL5 data was available at baseline for 60 patients, with no differences identified in patient characteristics between those with and without CCL5 data (Table 3).

**Table 3:** Patient characteristics for all patients randomised within the SCALOP trial subdivided by the availability of data relating to serum C-C chemokine ligand 5 (CCL5) quantification.

	CCL5 data (*n* = 60)		No CCL5 data (*n* = 14)		Total (*n* = 74)	
	*n*	%	*n*	%	*n*	%
Treatment						
Gemcitabine	30	50	6	43	36	49
Capecitabine	30	50	8	57	38	51
Sex						
Male	33	55	8	57	41	55
Female	27	45	6	43	33	45
Age (years)						
<65	33	55	5	36	38	51
≥65	27	45	9	64	36	49
WHO PS						
0	34	57	6	43	40	54
1	26	43	8	57	34	46
CA19–9 Median (IQR) *U/mL*	240.5 (77.0, 822.0)*		110.0 (71.0, 720.0)**		212.0 (73.0, 815.0)	
Disease diameter Median (IQR) *cm*	3.80 (3.00, 4.58)		4.35 (3.15, 4.95)		3.90 (3.00, 4.85)	

Disease diameter refers to the longest axis of the tumour.

*CA19–9* cancer antigen 19–9, *IQR* interquartile range, *n* number, *WHO PS* World Health Organisation Performance Status.

*n = 54.

**n = 13.

**Fig. 1:**
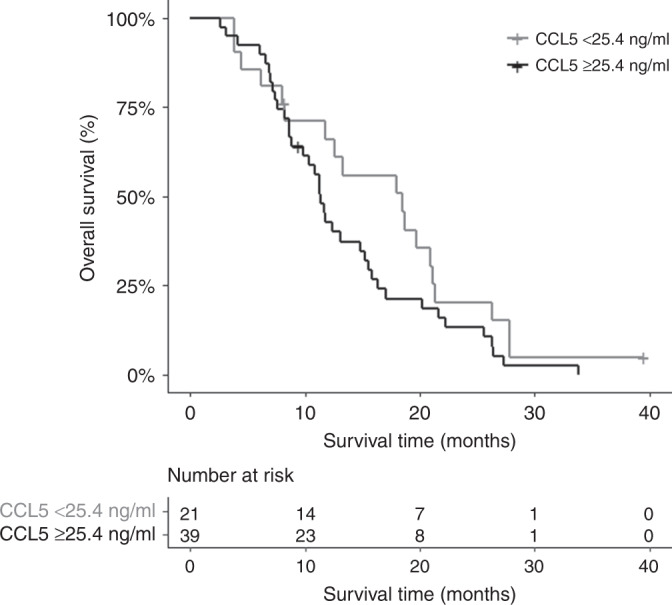
Kaplan–Meier estimates of overall survival for dichotomised serum C-C chemokine ligand 5 (CCL5) concentration in patients randomised within the SCALOP trial.

We identified a linear association of CCL5 with OS. When associated with OS in a multivariable Cox proportional hazards model as a continuous variable, patients with high circulating CCL5 were found to have a HR of 1.01 for each ng/ml unit increase (95% CI 1.00–1.03; *p* = 0.013, *n* = 54) (Table 4). A time-dependent ROC curve was constructed to identify the optimal threshold for CCL5 (Supplementary Fig. 2). Dichotomisation of CCL5 at its optimal threshold of 25.4 ng/ml was significantly associated with OS, with a HR of 1.95 (95% CI: 1.04–3.65; *p* = 0.037) in the Cox multivariable model. Median OS was 18.5 months in patients with CCL5^low^ (21/60) (95% CI: 11.76–21.32) and 11.3 months (95% CI: 9.86–15.51) in patients with CCL5^high^ (39/60); as demonstrated in Fig. 1. Diagnostic tests showed that there was no evidence of departure from proportionality or violations of the linearity assumption, and that there were no extreme outliers or influential points.

**Table 4:** Univariable and multivariable Cox regression analysis by characteristic.

	*n*	Univariable		Multivariable (*n* = 54)	
		HR (95% CI)	*p*-value	HR (95% CI)	*p*-value
Biomarker					
CCL5 (ng/ml)	60	1.01 (1.00–1.03)	0.013	1.01 (1.00–1.03)	0.011
CA19–9 (U/mL)	54	1.20 (1.11–1.31)	<0.001	1.19 (1.08–1.30)	<0.001
Age (years)					
<65	33	1.00		1.00	
≥65	27	0.83 (0.48–1.43)	0.503	0.69 (0.37–1.27)	0.234
Performance status					
0	34	1.00		1.00	
1	26	1.89 (1.09–3.27)	0.024	1.92 (0.96–3.84)	0.064
Trial arm					
Capecitabine	30	1.00		1.00	
Gemcitabine	30	1.25 (0.74–2.12)	0.409	0.98 (0.52–1.84)	0.951

Median CCL5 was 30.0 (IQR 6–48) ng/ml and median CA19–9 was 241 (IQR 77–822) U/ml. For multivariable analysis, six patients were missing cancer antigen 19–9 (CA19–9) data. Hazard ratios (HRs) for CCL5 (1.35) and CA19–9 (1.20) were calculated for each increase in CCL5 of 1 ng/ml and increase in CA19–9 of 1000U/ml, respectively.

*95% CI* 95% confidence interval, *HR* hazard ratio, *IQR* interquartile range, *n* number, *OS* overall survival.

CCL5 circulating levels were additionally assessed in the context of other clinicopathological characteristics, including age, PS and CA19–9 (*n* = 54 for CA19–9 data). A signature utilising dichotomised CCL5 was determined (Supplementary Fig. 3) including age, PS and CA19–9. The CCL5 signature was created for each patient using the beta coefficients from the multivariable survival model and multiplying them by their corresponding covariate (CCL5 × 0.6678 − Age × 0.4072 + WHO PS × 0.7807 + CA199 × 0.0001663). With the optimal threshold determined at 0.398, 17 patients were classified as CCL5^low^ and 37 as CCL5^high^. Using this approach, patients classified as CCL5^low^ had a median OS of 19.68 months (95% CI 16.3–27.79) and CCL5^high^ of 11.2 months (95% CI 8.25–13.24); hazard ratio 2.69 (95% CI 1.40–5.17; *p* = 0.003) (Supplementary Table 3).

Relative change in CCL5 levels between baseline (prior to start of GEMCAP) and week 17 (prior to start of CRT, data available for *n* = 47) did not demonstrate any association with OS (Supplementary Table 4). Independent measurement of CCL5 at week 17 did not associate with OS. High levels of circulating CCL5 were identified to be a poor prognostic factor independent of concurrent chemotherapy received during radiotherapy (capecitabine or gemcitabine). Taken together, these suggest that circulating CCL5 levels at baseline relate to intrinsic tumour properties. No benefit of CCL5 as a potential pharmacodynamic biomarker was identified within the context of the CRT treatment delivered in this randomised trial.

## Discussion

### Interpretation of results in the context of pre-specified hypotheses and other relevant studies

Pancreatic cancer responds poorly to chemotherapy and radiotherapy and the aim of this research was to determine the prognostic value of previously reported circulating biomarkers in patients with LAPC treated with chemotherapy and CRT. To the best of our knowledge, this is the first biomarker study from a prospective randomised clinical trial in LAPC. The cytokine panel tested in this study was based on a systematic review that identified cytokines of diagnostic, prognostic or predictive significance in PDAC.^10^ This included six cytokines (IL-1β; IL-6, IL-8, VEGF, TGFβ, IL-10) previously reported as consistently elevated in patients with PDAC compared to healthy controls, all of which have previously been found to have potential prognostic value (carrying higher risk of metastasis and lower OS).^10,13–19^ However, none of these six cytokines correlated with survival in the SCALOP trial.

We identified baseline levels of circulating CCL5 and IFNγ as being significantly associated with OS, of which CCL5 remained significant in multivariable analysis. CCL5 can be secreted by a variety of tumour cells (including pancreatic cancer) as well as non-malignant stromal cells including T regulatory cells (T_regs_) and macrophages. Engagement of CCL5 with its receptor, C-C chemokine receptor type 5 (CCR5), can favour tumour growth by several mechanisms including induction of the mammalian target of rapamycin (mTOR) pathway,^20,21^ and by recruiting tumour associated macrophages (TAMs), leading to immunosuppression and release of pro-angiogenic cytokines.^22,23^ The CCL5-CCR5 axis has also been implicated in tumour migration and metastasis through modulating the activity of the PI3K/Akt, MAPK/ERK and NF-ĸB pathways, and via induction of matrix metalloproteinases.^24^

The interaction between pancreatic tumour cells, the immune system and the role of the CCL5-CCR5 axis has been investigated in preclinical studies. Tan et al. demonstrated that both human pancreatic cancer and murine pancreatic tumour (Pan02) secreted CCL5 and correspondingly, within the stroma, CCR5 was preferentially expressed by CD4^+^ Foxp3^+^ T_regs_.^23^ The investigators also showed that T_reg_ infiltration within the tumour could be reduced by systemic administration of CCR5 antagonists, resulting in restricted tumour growth. CCL5 secretion in pancreatic cancer was further investigated by Wang et al in a cohort of 120 resected PDAC tissues. While Foxp3 is typically utilised to identify T_regs_, here the investigators demonstrated the presence of Foxp3 positive cancer cells. CCL5 was directly trans-activated by cancer-Foxp3, which in turn promoted T_reg_ infiltration.^24^ Intriguingly, Jang et al. reported accumulation of T_regs_ around murine pancreatic tumours within one week of implantation, suggesting tumour cell-intrinsic secretion of CCL5 plays an important role in this rapid infiltration.^25^ This proposes a model of cell intrinsic CCL5 secretion resulting in autocrine and paracrine signalling promoting a pro-tumourigenic immunosuppressive TME. However, the prognostic implication of circulating CCL5 has not been previously reported in literature.

### Implications for future research and clinical value of the work

PDAC has been described as an “immune desert” and conventional immunotherapy has failed to impact outcomes in this disease. Low mutational burden, induction of TAMs and myeloid-derived suppressor cells, and exclusion of CD8 + and other pro-inflammatory cells are some of the proposed mechanisms for failure of immunotherapy. Mitigating the factors that contribute to immune suppression may enhance response to immunotherapy in PDAC. Several strategies, including combinations of immunotherapy with gemcitabine, radiation, pancreatic cancer vaccine (GVAX) and CSF1R antibody, are being evaluated in clinical trials.^26,27^ The biomarker analysis of this clinical cohort is consistent with the pre-clinical hypothesis that the CCL5-CCR5 axis plays an adverse role in pancreatic cancer pathogenesis. Given its immunomodulatory effects, inhibition of the CCL5-CCR5 axis in combination with immunotherapy should be tested in pre-clinical models.

#### Discussion of limitations

We have been unable to validate a number of previously reported prognostic biomarkers in the prospective trial cohort reported here. It is unclear whether this relates to the trial cohort under study or to limitations in previous studies that identified these candidate biomarkers. In this study, we analysed biomarkers in patients randomised within SCALOP but not in 40/114 patients ineligible for randomisation due to disease progression, poor PS or because of patient or clinician choice. This may have biased the analysed cohort and validation of our findings in a larger cohort is required.

Although clear protocols for sample collection and processing were pre-specified, variations between centres is possible. For example, 18/27 centres did not have facilities or regulatory approvals for sample storage, thereby requiring unprocessed samples to be shipped centrally to WCB for processing. Given that this study predates the widespread adoption of preservative tubes that allow a 14-day window within which samples can be transported and processed, the requirement for many samples to be sent to WCB may have impacted on their quality. Nevertheless, all assays were performed under identical experimental conditions at a single centre using pre-defined protocols.

A final point of note is that the median biomarker levels we have reported here differ from their measurement in previous studies of pancreatic cancer.^17–19^ For example, the measured values for IL-6 and IL-8 are a factor of between 3 and 5 higher than in some previous reports. Levels of IL-1β were also higher here compared with previous reports (a median of 30 pg/ml compared with 0 pg/ml in the existing literature). The median CCL5 reading is also around 200-fold higher than for example reported previously in pancreatic cancer by Farren and colleagues,^19^ but at 30.1 ng/ml is similar in magnitude to values that have been reported for ovarian, breast and cervical cancer.^28,29^ These variations may reflect differences in the studied patient populations, in the storage and processing of samples, and in the assays used to process these samples. Nevertheless, they do not detract from the standardised conditions used to compare serum biomarker levels for patients within this study, from which we identified clinical support for pre-clinical studies that have previously postulated a role for the CCL5-CCR5 axis in the pathogenesis of pancreatic cancer.

## Conclusion

Circulating CCL5 is an independent marker for poor prognosis for patients with LAPC treated with combination chemotherapy and consolidation CRT within the SCALOP trial. Further studies are required to validate CCL5 as a tumour marker in LAPC. Blockade of the CCL5-CCR5 axis may provide opportunities to modulate the efficacy of immunotherapy in pancreatic cancer.

## Supplementary information

Supplementary Materials

## Data Availability

Anonymised data available on request from the corresponding author.

## References

[1] Rahib L, Smith B, Aizenberg R, Rosenzweig A, Fleshman J, Matrisian L (2014). Projecting cancer incidence and deaths to 2030: the unexpected burden of thyroid, liver, and pancreas cancers in the United States. Cancer Res..

[2] Conroy T, Hammel P, Hebbar M, Ben Abdelghani M, Wei AC, Raoul JL (2018). FOLFIRINOX or gemcitabine as adjuvant therapy for pancreatic cancer. N. Engl. J. Med..

[3] Hammel P, Lacy J, Portales F, Sobrero A, Pazo Cid R, Manzano, Mozo JL (2018). Phase II LAPACT trial of nab-paclitaxel (nab-P) plus gemcitabine (G) for patients with locally advanced pancreatic cancer (LAPC). J. Clin. Oncol..

[4] Bailey P, Chang DK, Nones K, Johns AL, Patch AM, Gingras MC (2016). Genomic analyses identify molecular subtypes of pancreatic cancer. Nature.

[5] Moffitt R, Marayati R, Flate E, Volmar K, Loeza S, Hoadley K (2015). Virtual microdissection identifies distinct tumor- and stroma-specific subtypes of pancreatic ductal adenocarcinoma. Nat. Genet..

[6] Collisson E, Sadanandam, Olson P, Gibb WJ, Truitt M, Gu S (2011). Subtypes of pancreatic ductal adenocarcinoma and their differing responses to therapy. Nat. Med..

[7] Golan T, Hammel P, Reni M, Van Cutsem E, Macarulla T, Hall MJ (2019). Maintenance Olaparib for germline BRCA-mutated metastatic pancreatic cancer. N. Engl. J. Med..

[8] Mukherjee S, Hurt CN, Bridgewater J, Falk S, Cummins S, Wasan H (2013). Gemcitabine-based or capecitabine-based chemoradiotherapy for locally advanced pancreatic cancer (SCALOP): a multicentre, randomised, phase 2 trial. Lancet Oncol..

[9] Hurt C, Falk S, Crosby T, McDonald A, Ray R, Joseph G (2017). Long-term results and recurrence patterns from SCALOP: a phase II randomised trial of gemcitabine- or capecitabine-based chemoradiation for locally advanced pancreatic cancer. Br. J. Cancer.

[10] Yako YY, Kruger D, Smith M, Brand M (2016). Cytokines as biomarkers of pancreatic ductal adenocarcinoma: a systematic review. PLoS ONE.

[11] McShane LM, Altman DG, Sauerbrei W, Taube SE, Gion M, Clark GM (2005). Statistics subcommittee of the NCI-EORTC working group on cancer diagnostics. REporting recommendations for tumour MARKer prognostic studies (REMARK). Br. J. Cancer.

[12] Byers LA, Holsinger FC, Kies MS, William WN, El-Naggar AK, Lee JJ (2010). Serum signature of hypoxia-regulated factors is associated with progression after induction therapy in head and neck squamous cell cancer. Mol. Cancer Ther..

[13] Torres C, Linares A, Alejandre MJ, Palomino-Morales RJ, Caba O, Prados J (2015). Prognosis relevance of serum cytokines in pancreatic cancer. Biomed. Res. Int.

[14] Błogowski W, Deskur A, Budkowska M, Sałata D, Madej-Michniewicz A, Dabkowski K (2014). Selected cytokines in patients with pancreatic cancer: a preliminary report. PLoS ONE.

[15] Delitto D, Black B, Sorenson H, Knowlton A, Thomas R, Sarosi G (2015). The inflammatory milieu within the pancreatic cancer microenvironment correlates with clinicopathologic parameters, chemoresistance and survival. BMC Cancer.

[16] Wörmann S, Diakopoulos K, Lesina M, Algül H (2014). The immune network in pancreatic cancer development and progression. Oncogene.

[17] Mitsunaga S, Ikeda M, Shimizu S, Ohno I, Furuse J, Inagaki M (2013). Serum levels of IL-6 and IL-1β can predict the efficacy of gemcitabine in patients with advanced pancreatic cancer. Br. J. Cancer.

[18] Carbone A, Vizio B, Novarino A, Mauri FA, Geuna M, Robino C (2009). IL-18 paradox in pancreatic carcinoma: elevated serum levels of free IL-18 are correlated with poor survival. J. Immunother..

[19] Farren MR, Mace TA, Geyer S, Mikhail S, Wu C, Ciombor K (2016). Systemic immune activity predicts overall survival in treatment-naïve patients with metastatic pancreatic cancer. Clin. Cancer Res..

[20] Murooka TT, Rahbar R, Fish EN (2009). CCL5 promotes proliferation of MCF-7 cells through mTOR-dependent mRNA translation. Biochem. Biophys. Res. Commun..

[21] Gao D, Cazares L, Fish E (2017). CCL5-CCR5 interactions modulate metabolic events during tumor onset to promote tumorigenesis. BMC Cancer.

[22] Argyle D, Kitamura T (2018). Targeting macrophage-recruiting chemokines as a novel therapeutic strategy to prevent the progression of solid tumors. Front. Immunol..

[23] Tan M, Goedegebuure P, Belt B, Flaherty B, Sankpal N, Gillanders W (2009). Disruption of CCR5-dependent homing of regulatory T cells inhibits tumor growth in a murine model of pancreatic cancer. J. Immunol..

[24] Wang X, Lang M, Zhao T, Feng X, Zheng C, Huang C (2017). Cancer-FOXP3 directly activated CCL5 to recruit FOXP3+Treg cells in pancreatic ductal adenocarcinoma. Oncogene.

[25] Jang JE, Hajdu CH, Liot C, Miller G, Dustin ML, Bar-Sagi D (2017). Crosstalk between regulatory T cells and tumor-associated dendritic cells negates anti-tumor immunity in pancreatic cancer. Cell Rep..

[26] Papadopoulos K, Gluck L, Martin L, Olszanski A, Tolcher A, Ngarmchamnanrith G (2017). First-in-human study of AMG 820, a monoclonal anti-colony-stimulating factor 1 receptor antibody, in patients with advanced solid tumors. Clin. Cancer Res..

[27] Le D, Picozzi V, Ko A, Wainberg Z, Kindler H, Wang-Gillam A (2019). Results from a Phase IIb, randomized, multicenter study of GVAX pancreas and CRS-207 compared with chemotherapy in adults with previously treated metastatic pancreatic adenocarcinoma (ECLIPSE Study). Clin. Cancer Res..

[28] Tsukhishiro S, Suzumori N, Nishikawa H, Arakawa A, Suzumori K (2006). Elevated serum RANTES levels in patients with ovarian cancer correlate with the extent of the disorder. Gynaecol. Oncol..

[29] Niwa Y, Akamatsu H, Niwa H, Sumi H, Ozaki Y, Abe A (2001). Correlation of tissue and plasma RANTES levels with disease course in patients with breast or cervical cancer. Clin. Cancer Res..

