# Supplementary material for: Circulating biomarkers and outcomes from a randomised phase 2 trial of gemcitabine versus capecitabine-based chemoradiotherapy for pancreatic cancer
Source: Br J Cancer. 2020 Oct 26;124(3):581–6. doi: 10.1038/s41416-020-01120-z (PMC7851394; doi:10.1038/s41416-020-01120-z)
Supplement: Supplementary file 1 — Supplementary Materials [file 41416_2020_1120_MOESM1_ESM.docx]

**SUPPLEMENTARY FILES**

**Supplementary tables**

|  | | **Biomarker Data Available** (n=63) | | |  | **No Biomarker Data** (n=11) | | |  | **Total** (n=74) | | |
| --- | --- | --- | --- | --- | --- | --- | --- | --- | --- | --- | --- | --- |
|  | | **n** | | **%** |  | **n** | | **%** |  | **n** | | **%** |
| **Treatment** | |  | | |  |  | | |  |  | | |
|  | Gemcitabine | 32 | 51 | |  | 6 | 55 | |  | 38 | 51 | |
|  | Capecitabine | 31 | 49 | |  | 5 | 45 | |  | 36 | 49 | |
| **Sex** | |  |  | |  |  |  | |  |  |  | |
|  | Male | 36 | 57 | |  | 5 | 45 | |  | 41 | 55 | |
|  | Female | 27 | 43 | |  | 6 | 55 | |  | 33 | 45 | |
| **Age** [years] | |  |  | |  |  |  | |  |  |  | |
|  | <65 | 35 | 56 | |  | 3 | 27 | |  | 38 | 51 | |
|  | ≥65 | 28 | 44 | |  | 8 | 73 | |  | 36 | 49 | |
| **Performance status** | |  |  | |  |  |  | |  |  |  | |
|  | 0 | 37 | 59 | |  | 3 | 27 | |  | 40 | 54 | |
|  | 1 | 26 | 41 | |  | 8 | 73 | |  | 34 | 46 | |
| **Disease diameter** [cm], (IQR) | | 3.8 (3.0, 4.8) | | |  | 4.2 (3.3, 5.3) | | |  | 3.9 (3.0, 4.9) | | |

**Supplementary Table 1. Patient characteristics for all patients randomised within the SCALOP trial by availability of biomarker data.** Disease diameter refers to the longest axis of the tumour. IQR, interquartile range; WHO PS, World Health Organisation Performance Status.

| **Biomarkers** | **Progressed** | **Not Progressed** | ***p-value*** | ***FDR*** |
| --- | --- | --- | --- | --- |
|  | Median (IQR) *pg/ml* | Median (IQR) *pg/ml* |  |  |
| bNGF | - | 6 (3, 14) | - | - |
| CCL11 | 153 (96, 189) | 161 (100, 252) | 0.5942 | 0.7475 |
| CCL2 | 178 (128, 282) | 195 (150, 298) | 0.4990 | 0.7475 |
| CCL27 | 706 (483, 869) | 621 (517, 751) | 0.3024 | 0.7475 |
| CCL5 | 29690 (15444, 39089) | 29402 (19493, 48992) | 0.5165 | 0.7475 |
| CCL7 | 105 (77, 145) | 114 (87, 142) | 0.6222 | 0.7475 |
| CD25 | 1216 (934, 1564) | 1105 (850, 1378) | 0.3288 | 0.7475 |
| CXCL1 | 139 (116, 162) | 161 (128, 214) | 0.4083 | 0.7475 |
| CXCL8 | 9 (8, 16) | 18 (10, 71) | 0.0348 | 0.6521 |
| CXCL9 | 666 (607, 729) | 681 (616, 767) | 0.5800 | 0.7475 |
| CXCL10 | 70 (43, 82) | 51 (38, 74) | 0.1574 | 0.7475 |
| G-CSF | 14 ( 10, 17) | 16 (12, 19) | 0.4085 | 0.7475 |
| HGF | 452 (265, 491) | 375 (171, 531) | 0.7276 | 0.8396 |
| ICAM | 861999 (797902, 1217948) | 859438 (710587, 1062396) | 0.5708 | 0.7475 |
| IFN | 66 (52, 79) | 71 (57, 84) | 0.5031 | 0.7475 |
| IGF-1 | 265 (180, 335) | 156 (84, 279) | 0.8164 | 0.8747 |
| IL-3 | 49 (41, 72) | 73 (34, 71) | 0.9782 | 0.9782 |
| IL-6 | 5.5 (5, 9.5) | 5.5 (4.5, 8) | 0.9075 | 0.9388 |
| IL-7 | 7 (5.5, 11.5) | 7.5 (6, 9) | 0.8004 | 0.8747 |
| IL-10 | 3.5 (3.5, 4.5) | 4 (3, 4.5) | 0.5894 | 0.7475 |
| IL-16 | 428 (148, 917) | 1059 (145, 3405) | 0.1420 | 0.7475 |
| IL-18 | 896 (310, 1402) | 992 (362, 3155) | 0.3933 | 0.7475 |
| IL-1BETA | 23 (17, 31) | 29 (19, 56) | 0.2085 | 0.7475 |
| IL-1RA | 2694 (970, 3197) | 4344 (969, 11352) | 0.1921 | 0.7475 |
| M-CSF | 492 (448, 752) | 561 (418, 1006) | 0.6229 | 0.7475 |
| MIF | 14985 (7374, 22596) | 23541 (6894, 44987) | 0.5895 | 0.7475 |
| PDGF | 2191 (1540, 4350) | 3188 (1963, 5300) | 0.4403 | 0.7475 |
| SCF | 108 (71, 117) | 91 (73, 106) | 0.2064 | 0.7475 |
| TRAIL | 69 (45, 88) | 57 (39, 86) | 0.3709 | 0.7475 |
| VCAM | 1445101 (1252282, 1665204) | 1289097 (1010007, 1595178) | 0.2114 | 0.7475 |
| VEGF-A | 34 (19, 51) | 62 (32, 117) | 0.0435 | 0.6521 |

**Supplementary Table 2: Association of each biomarker when quantified at baseline, prior to treatment, with progression-free survival.** Association with progression free survival was determined using univariable Cox regression. FDR, false discovery rate; n, number.

| **Dichotomised CCL5 Signature** | **Overall survival (months)** | |  | **Univariate** | |
| --- | --- | --- | --- | --- | --- |
|  | **n** | **Median (95% CI)** |  | **HR (95% CI)** | ***p-value*** |
| <0.398 | 17 | 19.68 (16.30 - 27.79) |  | - | - |
| ≥0.398 | 37 | 11.20 (8.25 - 13.24) |  | 2.69 (1.40 - 5.17) | 0.003 |

**Supplementary Table 3: Overall survival and univariate analysis of dichotomised C-C chemokine ligand 5 (CCL5) signature.** 95% CI, 95% confidence interval; HR, hazard ratio.

|  | **Overall survival (months)** | |  | **Univariate** | |
| --- | --- | --- | --- | --- | --- |
| **% Change in CCL5** | **n** | **Median (95% CI)** |  | **HR (95% CI)** | ***p-value*** |
|  | 47 | -6.7 (-354.6, 264.1) |  | 1.00 (1.00, 1.00) | 0.42- |

**Supplementary Table 4: Overall survival and univariate analysis of percentage change in C-C chemokine ligand 5 (CCL5) signature from pre-treatment baseline to week 17 (prior to consolidation chemoradiotherapy).** 95% CI, 95% confidence interval; HR, hazard ratio.

**SUPPLEMENTARY FILES**

**Supplementary figures**

**
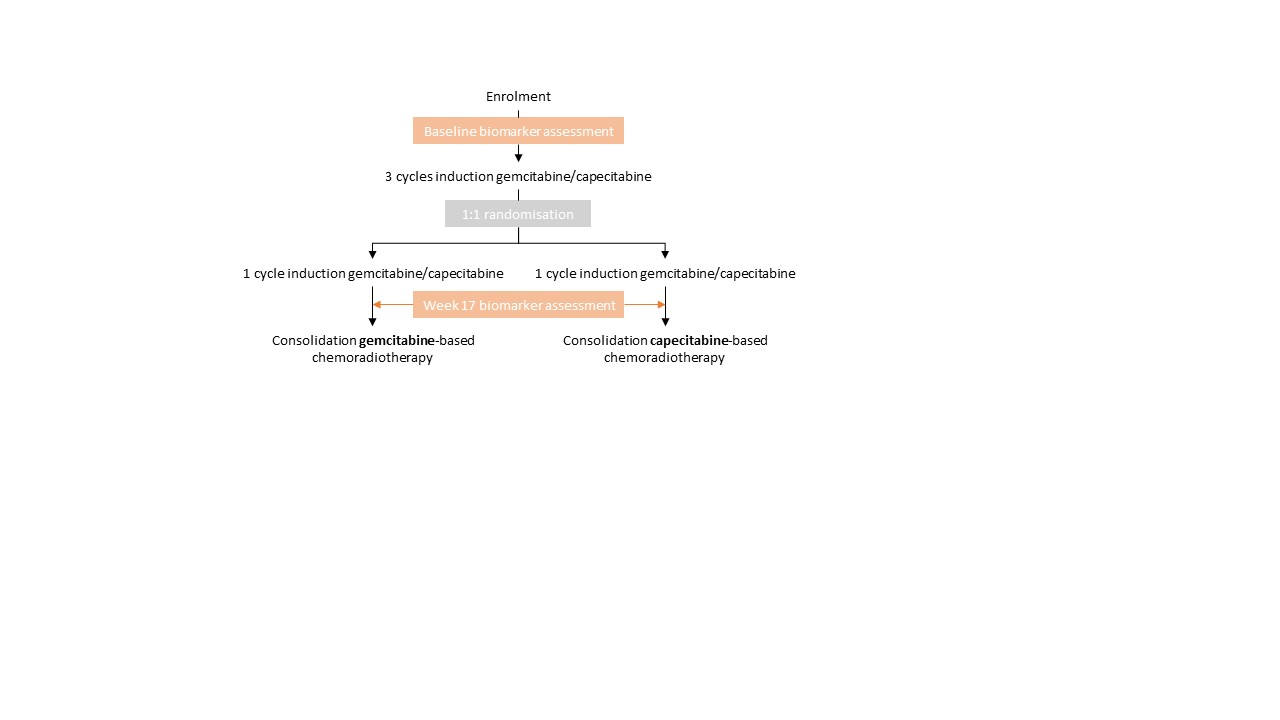
**

**Supplementary Figure 1: A schematic overview of the SCALOP trial, including key treatment timepoints.** Sample collection timepoints are highlighted in orange. Induction chemotherapy consisted of intravenous gemcitabine (1000mg/m^2^) on days 1, 8 and 15 of each 28 day cycle and oral capecitabine (830mg/m^2^ twice daily) on days 1-21 of each 28 day cycle. In total, four cycles of induction chemotherapy were administered prior to consolidation chemoradiotherapy at a total dose of 50.4Gy in 28 weekday fractions over 5.5 weeks with either intravenous gemcitabine (300mg/m^2^) weekly for six doses or oral capecitabine (830mg/m^2^) on each day radiotherapy was administered. Samples for biomarker analysis were collected prior to induction chemotherapy and prior to consolidation chemoradiotherapy.


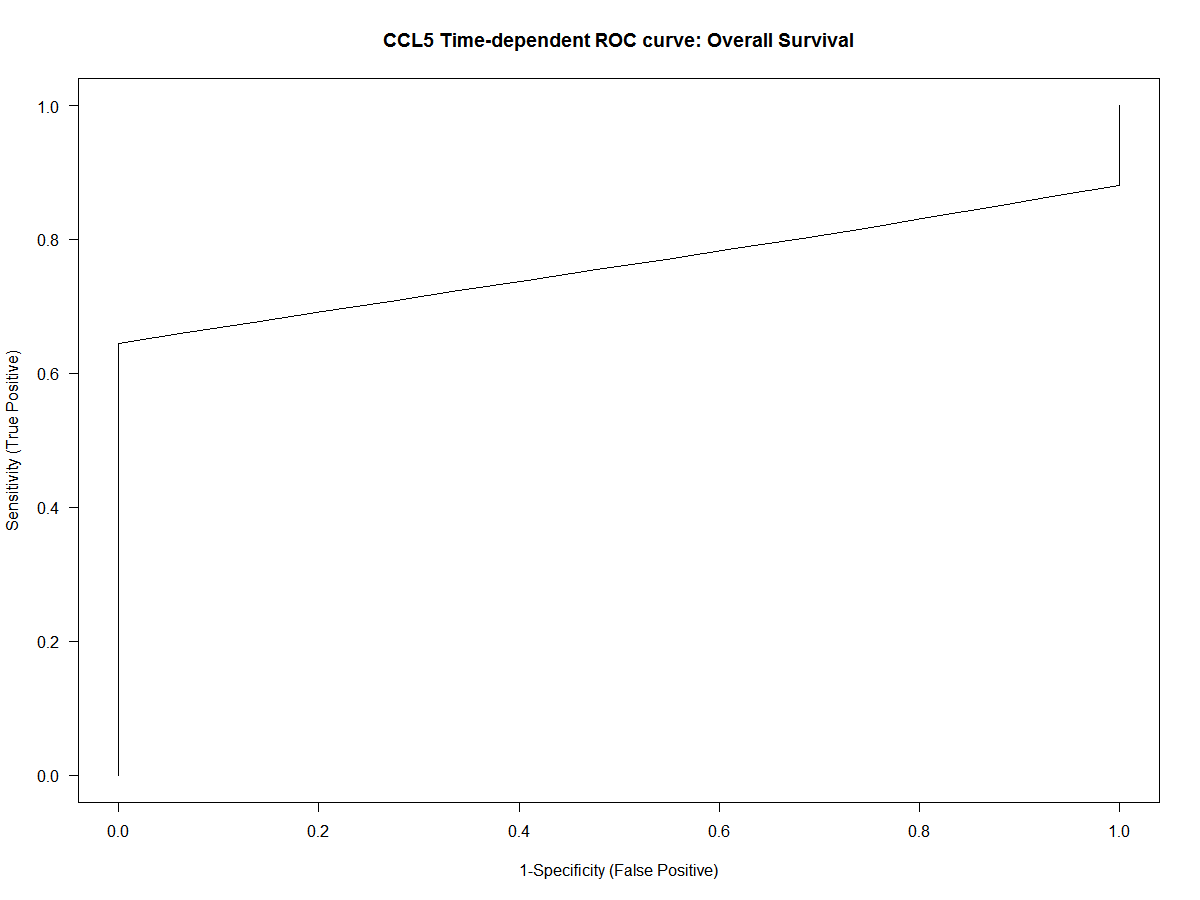


**Supplementary Figure 2: Time dependent receiver operating characteristic (ROC) curve estimation for circulating C-C chemokine ligand 5 (CCL5) association with overall survival.** Area under the curve (AUC): 0.761. Optimal cut point: 25.4 ng/ml. Sensitivity: 64%. Specificity: 100%. True positive: 64%. False positive: 0%.


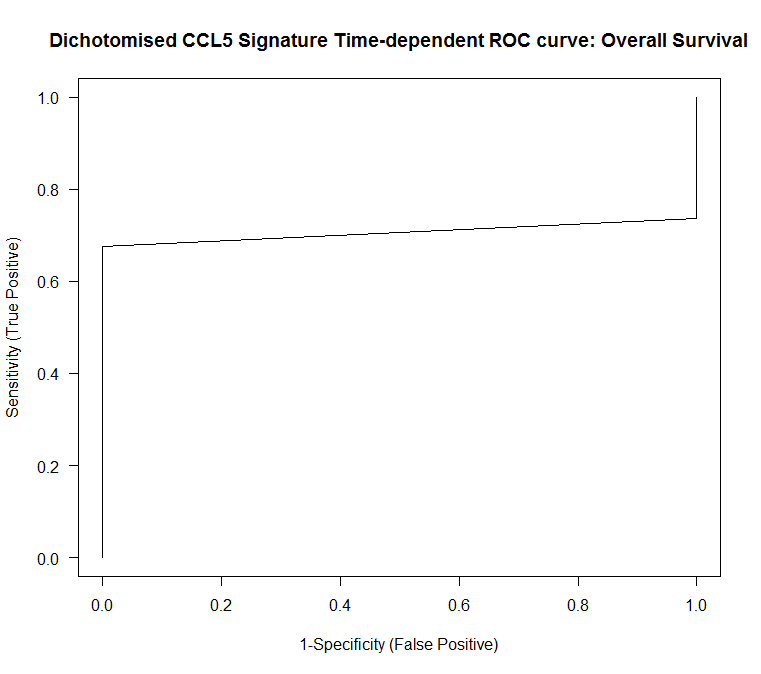


**Supplementary Figure 3: Time-dependent receive operating characteristic (ROC) curve estimation for a signature score including a dichotomised C-C chemokine ligand 5 (CCL5) association with overall survival.** Dichotomised CCL5 score was 0 if CCL5 < 25.4 ng/ml and 1 if CCL5 ≥ 25.4 ng/ml. Area under the curve (AUC): 0.707. Optimal cut point: 0.398. Sensitivity: 68%. Specificity: 100%. True positive: 68%. False positive: 0%.
